# Data on early postoperative changes in aqueous monocyte chemoattractant protein-1 levels after phacoemulsification

**DOI:** 10.1016/j.dib.2016.11.005

**Published:** 2016-11-09

**Authors:** Motofumi Kawai, Toshihiro Inoue, Akitoshi Yoshida, Hidenobu Tanihara

**Affiliations:** a Department of Ophthalmology, Asahikawa Medical University, Asahikawa, Japan; b Department of Ophthalmology, Faculty of Life Sciences, Kumamoto University, Kumamoto, Japan

**Keywords:** MCP-1, Phacoemulsification

## Abstract

The data presented in this article are related to the research article entitled “Elevated levels of monocyte chemoattractant protein-1 in the aqueous humor after phacoemulsification” (M. Kawai, T. Inoue, M. Inatani, N. Tsuboi, K. Shobayashi, A. Matsukawa, A. Yoshida, H, 2012) [1]. The mean (±SE) aqueous MCP-1 levels (pg/ml) were 31.2±12.5, 1931.2±910.7, 2172.2±1015.7, 3315.4 ±1535.8, 3015.9 ±914.4, 2709.0 ±738.7, 72.8 ±26.9, and 207.1±62.9 at 0, 3, 6, 12, 24, 48, 168, and 720 h after phacoemulsification, respectively. The immunohistochemical analysis showed a number of MCP-1 positive inflammatory cells in the anterior chamber and conjunctiva. There were some MCP-1 positive cells in the corneal endothelium.

**Specifications Table**

**Table:** 

Subject area	Biology
More specific subject area	MCP-1 after phacoemulsification
Type of data	Figures
How data was acquired	ELISA and Immunohistochemistry
Data format	Analyzed
Experimental factors	Aqueous levels of MCP-1 and localization of MCP-1 positive cells in anterior ocular segment
Experimental features	Time-dependent changes in aqueous MCP-1 level after phacoemulsification
Data source location	Kumamoto University, Kumamoto City, Japan
Data accessibility	The data are within this article

**Values of the data**

• Aqueous level of MCP-1 could be a reliable marker of ocular inflammation after phacoemulsification.
• The data are useful to understand the time-dependent changes in ocular inflammation after phacoemulsification.
• Methodology can be used for further estimation of other cytokine.

## 1 Data

**Fig. 1:**
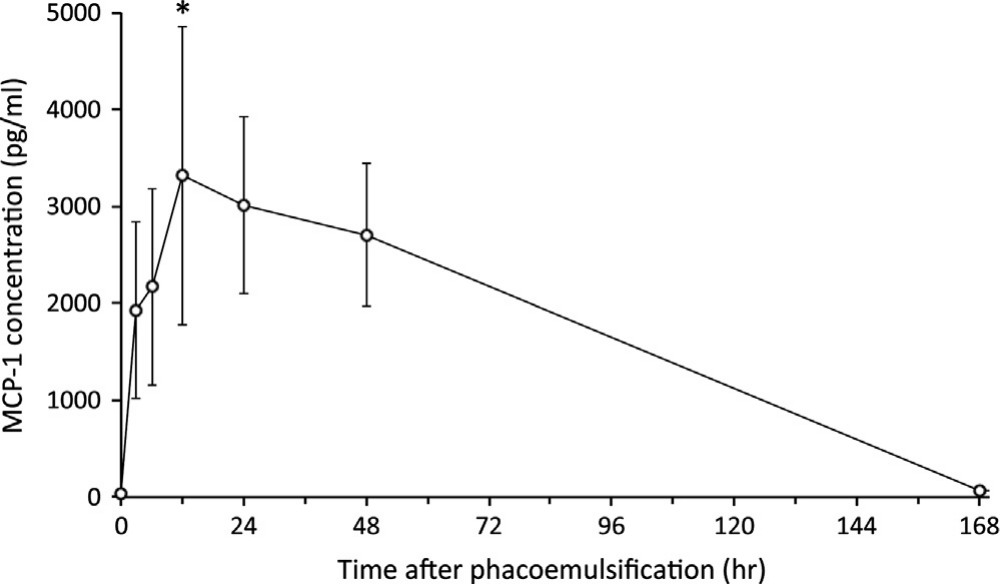
Time-dependent changes in MCP-1 levels after phacoemulsification. Mean±standard error, ^*^P<0.05 using Dunnett׳s test.

**Fig. 2:**
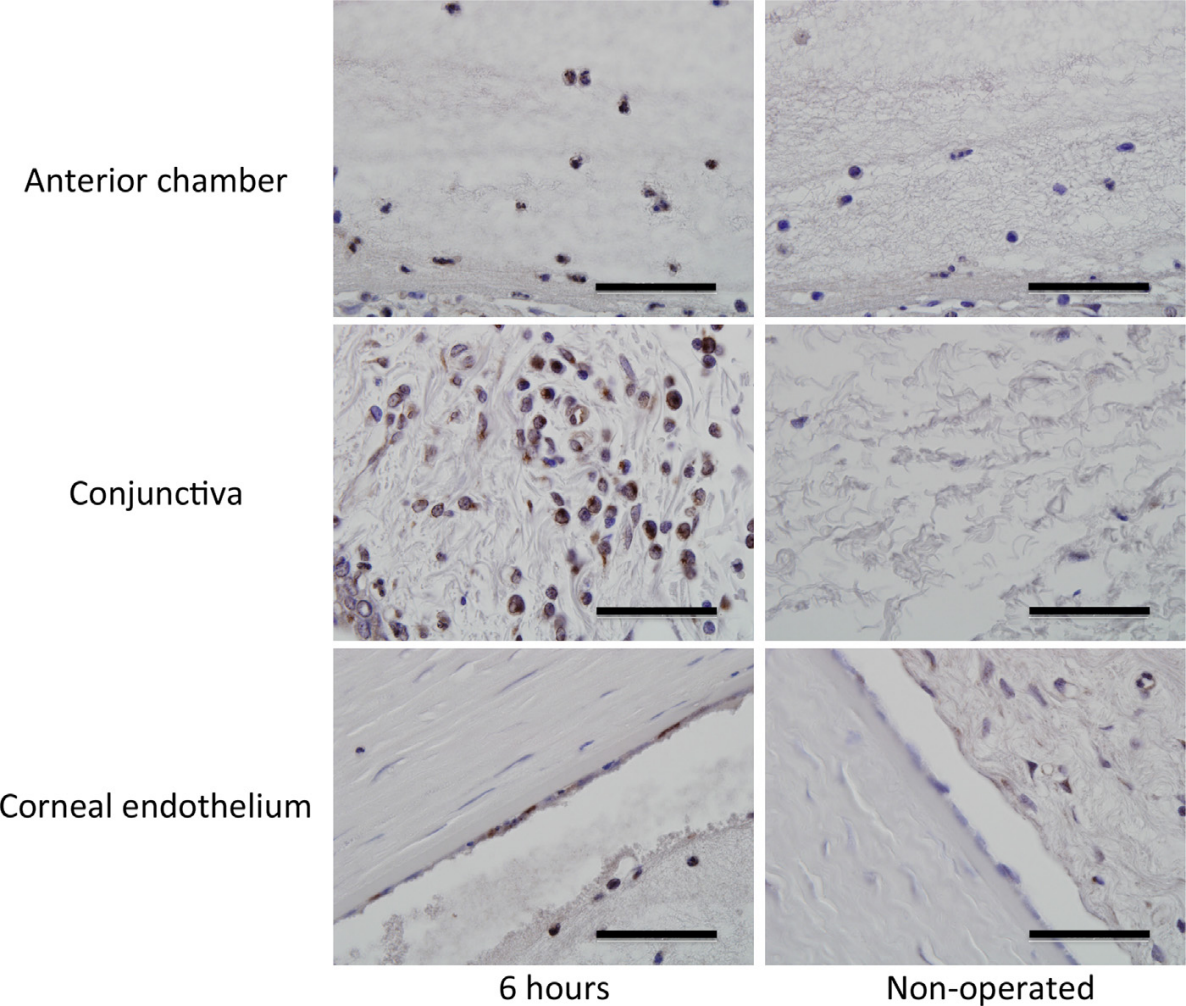
Immunohistochemical analysis for MCP-1 (brown) in anterior eye segments at 6 h after phacoemulsification. Hematoxylin was used as a counter stain. Scale bar, 50 µm.

Fig. 1 shows the time course of aqueous MCP-1 levels after phacoemulsification. Levels increased sharply with a peak at 12 h after surgery that gradually decreased for 7 days (168 h). It again increased for 30 days (720 h) as described in our previous report [1]. The mean (±SE) aqueous MCP-1 levels (pg/mL) were 31.2±12.5, 1931.2±910.7, 2172.2±1015.7, 3315.4±1535.8, 3015.9±914.4, 2709.0±738.7, and 72.8±26.9 at 0, 3, 6, 12, 24, 48, 168 h after surgery, respectively. The value at 12 h after surgery was significantly higher (*P*=0.041) than that of controls. The results of immunohistochemical analyses for MCP-1 are shown in Fig. 2. Six hours after phacoemulsification, many MCP-1-positive cells were found in the anterior chamber and conjunctiva, and MCP-1 immunoreactivities were noted in some corneal endothelial cells. In contrast, no MCP-1-positive cells were observed in control eyes.

## 2 Experimental design, materials and methods

The animals subjected to surgery were euthanized after collecting aqueous humor 3, 6, 12, 24, 48, 168, and 720 h after surgery (*n*=4–6), and six control animals were euthanized without surgery. ELISA and immunohistochemical analysis were conducted as described previously [1]. Dunnett׳s test was used for statistical analyses, and *P* values less than 0.05 were considered significant.

## Conflict of interest

The authors report no conflicts of interest. The authors alone are responsible for the content and writing of the paper.
